# “Unity and Struggle of Opposites” as a Basis for the Functioning of Synthetic Bacterial Immobilized Consortium That Continuously Degrades Organophosphorus Pesticides

**DOI:** 10.3390/microorganisms10071394

**Published:** 2022-07-11

**Authors:** Elena Efremenko, Nikolay Stepanov, Olga Maslova, Olga Senko, Aysel Aslanli, Ilya Lyagin

**Affiliations:** Chemical Faculty, Lomonosov Moscow State University, Lenin Hills 1/3, 119991 Moscow, Russia; na.stepanov@gmail.com (N.S.); olga.maslova.rabota@gmail.com (O.M.); senkoov@gmail.com (O.S.); ayselaslanli@mail.ru (A.A.); lyagin@mail.ru (I.L.)

**Keywords:** synthetic consortium, degradation, organophosphorus pesticides, immobilized cells, *N*-acyl homoserine lactone, lactonase activity

## Abstract

This work was aimed at the development of an immobilized artificial consortium (IMAC) based on microorganisms belonging to the Gram-positive and Gram-negative bacterial cells capable of jointly carrying out the rapid and effective degradation of different organophosphorus pesticides (OPPs): paraoxon, parathion, methyl parathion, diazinon, chlorpyrifos, malathion, dimethoate, and demeton-S-methyl. A cryogel of poly(vinyl alcohol) was applied as a carrier for the IMAC. After a selection was made between several candidates of the genera *Rhodococcus* and *Pseudomonas*, the required combination of two cultures (*P. esterophilus* and *R. ruber*) was found. A further change in the ratio between the biomass of the cells inside the granules of IMAC, increasing the packing density of cells inside the same granules and decreasing the size of the granules with IMAC, gave a 225% improvement in the degradation activity of the cell combination. The increase in the velocity and the OPP degradation degree was 4.5 and 16 times greater than the individual *P. esterophilus* and *R. ruber* cells, respectively. Multiple uses of the obtained IMAC were demonstrated. The increase in IMAC lactonase activity confirmed the role of the cell quorum in the action efficiency of the synthetic biosystem. The co-inclusion of natural strains in a carrier during immobilization strengthened the IMAC activities without the genetic enhancement of the cells.

## 1. Introduction

It is known that organophosphorus pesticides (OPPs) continue to be actively used in the world as an effective means of protecting plants and animals. They are found in different water sources, thus creating the possibility of a serious impact on existing natural and artificial ecosystems [1,2]. Certain requirements are imposed on the concentrations of OPP, the presence of which is permissible in various environmental objects [3]. At the same time, these concentrations are strictly controlled by various analytical methods, which lead to the need for practical solutions to issues of compliance with established norms (http://www.fao.org/fao-who-codexalimentarius, accessed on 10 July 2022).

Most significantly, these problems of OPP removal concern aquatic environments due to the possible rapid migration of these pollutants together with water resources and the spread of pollution over large areas, including through atmospheric phenomena [4]. This problem of OPP removal from water sources is more acute as compared to soils since pesticides can remain in the soil in a bound state for a long time, for example, with humic substances [5,6]. This makes the search for a solution of this problem relevant for water systems.

One of the possible solutions for OPP removal from water sources is the use of microbial consortia that are not isolated from natural sources but artificially created, so-called synthetic consortia. The issues of creating easily reproducible artificial consortia based on the use of well-studied cell strains with stable, safe, and efficient functioning, in case of their application in practice for the sustainable development of ecological systems, are of great scientific and practical importance since they are components of the rational use of biological resources and actively developing synthetic biology [7,8]. The rational selection of microbial candidates and the experimental confirmation of the choice are often the drivers of such a process [9,10].

One of the effective approaches to creating artificial consortia that ensure the long-term and successful destruction of various pollutants is the use of microbial cells, in particular, bacteria in the form of highly concentrated cell populations. It is known that an increase in cell concentrations in such bacterial populations definitely leads to the activation of systems of programmed changes in the genetic and biochemical status of cells, leading to the formation of a quorum sensing response (QS) as a mechanism ensuring the stability of the functioning of such systems [11,12].

The possible successful immobilization of bacterial cells using various carriers to maintain concentrated cell populations in a stable form, allowing them to provide a high level of metabolic activity, has been repeatedly shown [13,14]. This is an extremely important finding, especially when the cells are used for the treatment and purification of various water systems from pollutants. Ideally, highly porous gel matrices are suitable for the implementation of this approach, providing mass transfer processes with minimal diffusion problems [15,16], in particular, cryogel of poly(vinyl alcohol) (PVA cryogel) [17,18].

Previously, we found that *Rhodococcus erythropolis* AC-1514D and *R. ruber* AC-1513D cells, known as active natural oil-oxidizing destructors, have the ability to catalyze the hydrolysis of five OPPs (paraoxon, malathion, demeton-S, diazinon, and dimethoate) [19], which in turn are characterized not only by an inhibitory effect on cholinesterases [20] but also on decarboxylases [21], causing cumulative damage to the human nervous system and triggering the development of various neurodegenerative diseases. 

The ability of *Rhodococcus* cells to destroy OPP is predetermined by the fact that these cells contain lactonase (EC 3.1.1.81), hydrolyzing various *N*-acyl homoserine lactones (AHL) produced by Gram-negative (G(−)) bacteria as signal molecules of their QS. However, lactonase has a high homology with organophosphorus hydrolase (OPH, EC 3.1.8.1), directly hydrolyzing OPP, but the catalytic activity of *Rhodococcus* lactonase in reaction with OPP is significantly lower in comparison with OPH due to the differences between the structures of the substrate-binding domains of the enzymes [19]. A number of other enzymes can catalyze the degradation of OPP [22,23], but the expediency of using whole living cells of microorganisms is fully justified for the subsequent disposal of OPP destruction products [24].

The tasks of deep OPP decomposition in nature are most effectively implemented by microbial consortia with complex cell compositions. In these consortia, as a rule, the metabolic products of one culture are substrates for another, or cells of different microorganisms oppose each other in competition for the same substrates, leading to the acceleration and deepening of the pollutants’ destruction [25]. In the case of OPP, the participants of such consortia are often *Pseudomonas* cells, which have the ability to destroy not only the pesticide molecules themselves but also their products [26,27]. Due to these findings, these bacteria are attractive for use in the development of synthetic consortia as well as *Rhodococcus* cells.

This work aimed to create an artificial consortium based on microorganisms belonging to the Gram-positive (G(+)) and G(−) bacterial cells capable of jointly carrying out the rapid and effective decomposition of different OPPs. To stabilize the degradation activity of such a synthetic consortium, pregrown and concentrated cells were immobilized in PVA cryogel by their joint entrapment to the same polymeric granules, and the further appearance of a synergistic effect between the microorganisms in this immobilized artificial consortium (IMAC) was investigated.

## 2. Materials and Methods

### 2.1. Chemicals

Parathion, methyl parathion, diazinon, chlorpyrifos, malathion, dimethoate, and demeton-S-methyl were purchased from Supelco (Bellefonte, PA, USA). Poly(vinyl alcohol) 16/1 (84 kDa) was purchased from Sinopec Corp. (Beijing, China). Paraoxon, *p*-nitrophenol, *N*-homoserine lactone hydrochloride, *N*-butyryl-D,L-homoserine lactone, *N*-(3-oxooctanoyl)-L-homoserine lactone, *N*-(3-oxodecanoyl)-L-homoserine lactone, *N*-(3-oxododecanoyl)-L-homoserine, and other chemicals were acquired from Sigma-Aldrich (St. Louis, MO, USA).

### 2.2. Microorganisms and Their Cultivation

The following strains of microorganisms were used in the work: *Rhodococcus erythropolis* AC-1514D, *R. ruber* AC-1513D [28], and *Pseudomonas esterophilus* V-1436D obtained from the All-Russian Collection of Microorganisms (VKM, Moscow, Russia, http://www.vkm.ru/, accessed on 10 July 2022) as well as *Pseudomonas* sp. 78G obtained from the laboratory of Ecobocatalysis of Lomonosov Moscow State University [29].

To accumulate cell biomass of *R. erythropolis* AC-1514D and *R. ruber* AC-1513D, the following medium (pH 7.0) was used for both cultures (g/L): glucose—10.0; yeast extract—1.0; Na citrate—5.0; KH_2_PO_4_—0.5; K_2_HPO_4_·3H_2_O—1.0; NaH_2_PO_4_·2H_2_O—0.5; (NH_4_)_2_SO_4_—1.0; MgSO_4_·7H_2_O—0.1; CaCl_2_·6H_2_O—0.05; FeSO_4_·7H_2_O—0.02. pH was adjusted by the addition of Na_2_CO_3_. 

To accumulate cell biomass of *P. esterophilus* V-1436D and *Pseudomonas* sp. 78G, the following medium (pH 7.0) was used for both cultures (g/L): trypton—10.0; yeast extract —5.0; NaCl—10.0. Bacteria were cultivated in 200 mL of medium in Erlenmeyer flasks (750 mL) on a shaker (Adolf Kuhner AG, Birsfelden, Switzerland) under aerobic conditions with constant agitation (180 rpm) at 28 °C. Cells were separated from the medium by centrifugation at 12,000× *g* for 15 min (Beckman-Coulter Inc., Fullerton, CA, USA) and used for their immobilization.

### 2.3. Cell Immobilization and Use of IMAC in Degradation Processes

The biomass precipitate was thoroughly mixed with a 10% (*w*/*w*) aqueous solution of PVA to obtain a 10–30% (*w*/*w*) concentration of bacterial cells, as described earlier [29,30]. In the composition of the resulting mixture of biomass of different cells, their weight ratios were varied. To form granules of different weights and sizes (from 50 ± 2 mg to 250 ± 5 mg), this mixture was pipetted into 96-well microplates, which were placed in a freezer at −20° C for 24 h and then thawed.

In a minimal medium of a certain composition (CaCl_2_·6H_2_O—0.01 g/L, MnSO_4_·7H_2_O—0.02 g/L, Na_2_HPO4—1.5 g/L, KH_2_PO_4_—1.0 g/L, MgSO_4_—0.2 g/L, NH_4_Cl—2.0 g/L, NaCl—5.0 g/L, pH 7.0) containing OPP or *p*-nitrophenol (0.15 mM), IMAC or immobilized cells of individual cultures were placed in such a way that the total concentration of wet cell biomass was always 20 g/L. The cell moisture content (%) was controlled by drying several samples of the precipitated bacterial cells to a constant weight.

The degradation of pollutants was carried out at room temperature. In experiments with multiple uses of IMAC, the duration of each working cycle was 24 h. At the end of each experiment, samples of the media were taken for analysis for the residual content of OPP or *p*-nitrophenol in the reaction medium. The pH values of the media were controlled at the end of cell cultivation. Potentiometric measurements were conducted to control the pH values of the prepared media and of the samples selected in the experiments; a Corning Pinnacle 530 pH meter (Corning Incorporated, New York, NY, USA) was used for this purpose.

To make sure that the PVA cryogel itself and the cells did not absorb pollutants but their biodegradation occurred, control studies were conducted in parallel, in which empty PVA cryogel granules without cells and with dead cells were exposed under the same conditions as living immobilized cells, and then these control samples were washed with a physiological solution in which the presence of sorbed pollutants was analyzed, and we took these data into account in the final calculations for the biodegradation of pollutants.

For multiple uses of IMAC under the batch conditions, the medium in the bioreactor was replaced with fresh sample after each working cycle, as described previously [31]. The granules of IMAC were washed with a sterile 0.9% (*w*/*v*) NaCl solution to remove the spent medium residues before loading a new medium portion to the reactor with immobilized cells.

To determine the dry weight, we used a sample of the biomass that was separated from the culture liquid by centrifugation (8000 rpm, 10 min, Avanti J 25 centrifuge, Beckman Coulter, Brea, CA, USA) and was brought to constant weight by drying at +80 °C. A granule of the immobilized biocatalyst was also dried to a constant weight. Knowing the initial concentrations of cells and the weight of the polymer when forming the granules, we calculated the concentrations of cells in the immobilized biocatalyst using the dry weight parameters.

### 2.4. Analytical Methods

#### 2.4.1. Determination of the Lactonase Activity of *R. ruber* Cells and IMAC

The lactonase activity of cells was determined by colorimetry (Agilent UV-853 spectrophotometer, Agilent Technologies, Waldbronn, Germany) concurrently using chromatographic and pH-sensitive indicator methods (Cresol Red, Sigma, USA) [19,32,33]. In this work, the following N-acyl homoserine lactones were used as substrates: *N*-homoserine lactone hydrochloride, *N*-butyryl-D,L-homoserine lactone, *N*-hexyl-L-homoserine lactone, *N*-(3-oxo-octyl)-L-homoserine lactone, *N*-(3-oxo-decyl)-L-homoserine lactone, and *N*-(3-oxo-dodecanoyl)-L-homoserine lactone.

The lactonase activity of *R. ruber* AC-1513D cells and IMAC was determined by a pH-sensitive indicator method as follows: The samples of the immobilized cells were placed in 945 μL of bicine buffer (pH 8.2; 0.625 mM) containing 0.1 M NaCl to which 5 μL of Creosol Red stock solution in DMSO (12.5 mM) and 50 μL of aqueous stock solution of an *N*-acylhomoserine lactone were added. The total volume of the reaction mixture was 1 mL. Spectrophotometric measurements (OD_570_) of the reaction mixture were performed every 15 min.

Additionally, the conversion of AHL molecules under the action of cells was analyzed by a chromatographic method according to the previously described technique [33] with some changes: First, 10 mL of AHL extracts were introduced into the column for reverse-phase chromatography. Mobile phases A and B were H_2_O and methanol, respectively, containing 0.1% glacial acetic acid. The elution started with 10% of mobile phase B for 5 min, and then it increased to 90% for 30 min, and remained at 90% for 15 min. The flow rate, column temperature, and wavelength of UV detection were 200 µL/min, 30 °C, and 210 nm, respectively.

The unit of cell activity was defined as the enzyme concentration in dried cells that brought about the hydrolysis of 1 μmol of substrate per minute at 25 °C (pH 8.2).

#### 2.4.2. Chromatographic Detection of the Pollutants

Dichloromethane (5 mL) was added to samples (5 mL) of media containing pollutants (paraoxon, diazinon, demethone-S-methyl, dimethoate, malathion, parathion, methyl parathion, and chlorpyrifos), and pesticide extraction was carried out. The determination of *p*-nitrophenol was carried out without preliminary extraction. Extracts were analyzed using HPLC (Knauer Smartline Pump 1000, Knauer, Berlin, Germany) on a Diasfer 110-C18 column for reverse-phase chromatography (Biochemmack ST, Moscow, Russia) (4.0 × 250 mm^2^) with a spectrophotometric detector and isocratic elution. 

A mixture of acetonitrile was used as eluent:water (60:40). The retention time was 4.5 min for paraoxon (OD_274_), 26.2 min for diazinon (OD_225_), 6.4 min for demethone–S-methyl (OD_210_), 3.4 min for dimethoate (OD_210_), 11.4 min for malathion (OD_210_), 31 min for chlorpyrifos (OD_225_), and 3.5 min for *p*-nitrophenol (OD_314_). The velocity of the eluent was 1 mL/min, and the temperature of the detector cell was 25 °C. The volume of the injected sample was 20 µL.

#### 2.4.3. Determination of ATP Concentrations in Microbial Cells

The cell viability and metabolic activity of all the immobilized cells used in this work were estimated by a bioluminescent luciferin–luciferase method that detected the concentration of adenosine triphosphate (ATP), as described previously [30]. For this purpose, granules with cell biomass were transferred to dimethyl sulfoxide (1 mL) and incubated at 25 °C for 1 h to extract the intracellular ATP. 

The luminescence of the samples was registered by a 3560 microluminometer (New Horizons Diagnostics Co., Columbia, MD, USA) with a luciferase reagent (Lyumtek OOO, Moscow, Russia). The correct concentration of ATP in all analyzed samples was estimated using calibration curves plotted with solutions of ATP standards.

### 2.5. Calculations of Process Characteristics and Statistical Analysis

The following characteristics of the process of pollutant (paraoxon or *p*-nitrophenol) degradation were calculated:

V_0_—the initial rate of pollutant degradation (µM/h). To calculate the initial rate of the biodegradation of pesticides, the process that occurred during the first 12 h was described by a first-order function (Ct = Co × e^−kt^).

De—the experimental degree of degradation (%) was calculated using the following approach:

De (%) = 100 − 100 × (C_pol_ (µM)/C_pol 0_ (µM)) (1)

where C_pol 0_ (µM) is the initial concentration of the pollutant (0.15 mM). 

C_pol_ (µM) is the residual concentration of the pollutants in the medium after its treatment with cells for 24 h.

Dt—the theoretical degree of degradation (%):

Dt (%) = Σ (De_i_ × W_i_) (2)

W_i_ = C_i_/C (3)

where i = 1 … n, and n is a participant of the created consortium; 

W_i_—the ratio of a certain participant in the mass cell content of the participant between the other participants in the consortium; 

C_i_ (g_wet cells_/L)—the concentration of the participant in the consortium, present in the reaction medium; 

C(20 g_wet cells_/L)—the concentration of consortium in reaction medium; 

ID—the improvement of degree degradation:

(%) = De (%) − Dt (%)(4)

was estimated for paraoxon or *p*-nitrophenol correspondently.

The data are shown as means of at least three independent experiments ± standard deviations (±SD). The statistical analysis was realized using SigmaPlot 12.5 (ver. 12.5, Systat Software Inc., San Jose, CA, USA). The significant (*p* ≤ 0.05) differences between the obtained results were estimated by a one-way analysis of variance (ANOVA).

## 3. Results

### 3.1. Selection of Cells for the Synthetic Consortium Capable of Degrading OPPs Such as Paraoxon and Product of Its Degradation, p-Nitrophenol

Since, as noted above, the bacterial cells of *R. erythropolis* AC-1514 D, *R. ruber* AC1513D, *Pseudomonas* sp. 78G, and *P. esterophilus* are capable of degrading OPP, it was decided to use them in combination in order to create an artificial consortium designed to purify aqueous solutions containing OPP. Paraoxon, the most commonly used pesticide for in vitro investigations, and the product of its hydrolysis, *p*-nitrophenol, were selected at this stage of research when screening among suitable bacterial combinations for the consortium was undertaken. It is worth noting that the joint use of microorganisms originally taken into work should have helped to identify the presence of possible patterns in the interactions between G(−) and G(+) bacterial cells in similar microbial biosystems.

The most useful composition of the consortium was determined using all four cultures. At the same time, all tested combinations of microorganisms, as well as individual cultures, were immobilized in a PVA cryogel in order to create equally high concentrations of cells (10% (*w*/*w*)) in the polymer granules and keep them in this state in the volume of a porous carrier to ensure the stability of the created biocatalytic systems.

IMAC was composed as granules containing *Pseudomonas* sp. 78G, *P. esterophilus*, *R. erythropolis* AC-1514D, and *R. ruber* AC-1513D bacterial cells immobilized in PVA cryogel. The cells were introduced to the carrier as biomass mixtures taken in various mass combinations within the same total concentration of cells loaded to the medium (20 g wet/L). These ratios between cell biomass are presented in Table 1 using different colors corresponding to certain microorganisms. The ratios ranged from 1:0 for individual cultures to 1:1, 1:1:1, and 1:1:1:1 when two, three, or four cell types were introduced into the IMAC, respectively. To determine the OPP-degrading ability, the created IMAC were injected into a medium containing 0.15 mM paraoxon. Such screening conditions were chosen based on the concentrations of OPP to be controlled [22] and the concentrations of cells often used when working with immobilized bacterial cells [34,35].

During these studies, the conversion rates of paraoxon and *p*-nitrophenol were compared, as well as the values of the so-called theoretical and experimental degradation of these compounds that were achieved with different variants of immobilized cells.

The screening of the potential composition of IMAC showed that the greatest degree of degradation of the pesticide (84.5%) was possessed by IMAC number 8, compiled on the basis of *R. ruber* and *P. esterophilus* cells (Table 1). At the same time, for this IMAC, the maximum increase in the observed degree of degradation was obtained (by 30%) compared to the expected value calculated theoretically, based on the results obtained using 100% cells of the individual cultures and taking into account the concentrations used for their introduction into the IMAC. The significant excess of the experimental values of the degree of degradation of the main pollutants studied over the expected values indicated a clear synergy and the appearance of not just an association of cells but a consortium.

Thus, it is obvious that the cells of *R. ruber* and *P. esterophilus* bacteria, being in a single pellet of PVA cryogel and in the corresponding reaction medium, exerted such an influence on each other, which resulted in the degree of degradation of paraoxon increasing when they were used together.

In addition, the initial rate of degradation of paraoxon, determined experimentally for the same IMAC (Table 1), turned out to be 1.2 times higher than the theoretically determined value: Vo,t = Vo (*P. esterophilus*) + Vo (*R. ruber*), where Vo,t is the theoretically calculated initial rate of decomposition of paraoxon by an immobilized consortium under conditions of 20 g cells/L and 0.15 mM pesticide; Vo (*P. esterophilus*) is the experimentally determined initial rate of degradation of paraoxon by immobilized *P. esterophilus* cells (10 g cells/L and 0.15 mM pesticide); and Vo (*R. ruber*) is the experimentally determined initial rate of degradation of paraoxon by immobilized *R. ruber* cells (10 g cells/L, 0.15 mM pesticide).

It is also worth noting that the IMAC obtained on the basis of *Pseudomonas* sp. 78G and *R. ruber* AC-1513D cells (sample number 10 in Table 1) also showed an increase in the experimental degree of paraoxon degradation compared to the theoretically calculated value. However, it was 19% and was lower than the result that was observed for the variant “*P. esterophilus* + *R. ruber*”.

A separate experiment to study the degradation of *p*-nitrophenol showed (Table 1) that the greatest increase in the degree of degradation compared to the calculated value (it was conducted similarly to the theoretical calculation for paraoxon) was obtained for a variant also consisting of *R. ruber* and *P. esterophilus* cells. The degree of degradation of *p*-nitrophenol under the action of this combination of cells increased by 2.2 times compared to the sum of the expected conversion values established for the same individual cultures. These data also confirmed the fact that the obtained result is a clear manifestation of a well-established consortium.

Thus, the consortium obtained on the basis of *P. esterophilus* and *R. ruber* bacterial cells was chosen for further research since it turned out to be the most effective according to the characteristics of the paraoxon degradation process and its decomposition product, *p*-nitrophenol.

### 3.2. Variation in the Ratio of Cells within the IMAC Based on P. enterophilus and R. ruber

**Figure 1:**
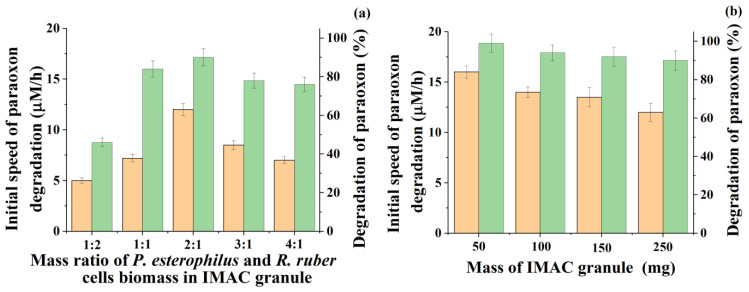
Influence of the ratio between *Pseudomonas* and *Rhodococcus* cell biomass in the content of IMAC (**a**) and the mass of each granule (**b**) with 10% (*w*/*w*) of total cell concentration on the initial speed of paraoxon (0.15 mM) degradation (■) and the degradation degree of the pesticide (■) catalyzed by the IMAC.

It was interesting to investigate the possibility of improving the characteristics of the two-component IMAC detected during the screening of four cultures by changing the ratio of cells inside it in the absence of a change in the total concentration of cells in the PVA cryogel granules and the reaction medium. The total concentration of cells in the granules, as before, was 10% (*w*/*w*) of the total mass of the biocatalyst. As expected, as a result of such a “probing” of the biosystem, a greater synergistic effect could be revealed in the process of paraoxon degradation due to the mutual influence of *P. esterophilus* and *R. ruber* cells. At the same time, the initial rate of degradation and the degree of degradation of the model pesticide remained the main parameters for control (Figure 1a).

An increase in the proportion of *Pseudomonas* cells in the IMAC composition provided a clear positive effect. Thus, the obtained results showed that by varying the mass ratio of cells during their immobilization into one granule, the initial rate of degradation of the pesticide can be increased by 66% in the most successful variant. In particular, for immobilized cells of *P. esterophilus* and *R. ruber* introduced into the PVA cryogel granule in a mass ratio of 1:1, the initial velocity was 7.2 ± 0.2 µM/h, and with the immobilization of the same cells in a ratio of 2:1, it increased to 12 ± 0.3 µM/h. This initial rate of paraoxon degradation turned out to be the highest among all tested variants of cell ratios. Along with an increase in the rate, there was also an increase in the degree of degradation of paraoxon during the time allotted for the process under study (24 h) (Figure 1a).

Apparently, with such a ratio inside the PVA cryogel granule, the competition between representatives of different cultures for the substrate was more intense inside the IMAC, which provided the best characteristics of the process (the speed and completeness of paraoxon degradation).

However, such an IMAC with a variant of the mass ratio of *P. esterophilus* and *R. ruber* bacterial cells equal to 2:1 and immobilized in one cryogel PVA granule was injected into a medium with paraoxon in the same total concentration and the same total number of cells inside the granules (10% (*w*/*w*) of the granule mass), but the size of the granules was reduced technologically to an acceptable level, which was 50 mg (Figure 1b). The subsequent use of the same IMAC, in the form of granules with a mass five times smaller, for the degradation of paraoxon showed the success of the actions taken since this allowed the process to be intensified by removing some obvious diffusion and mass transfer restrictions for the biosystem under study. As a result, it made it possible to increase the initial rate of the paraoxon process by 35% (up to 16.2 ± 0.3 µM/h) and to achieve 100% degradation of the pesticide

### 3.3. Multiple Uses of Developed IMAC for the Paraoxon Degradation

**Figure 2:**
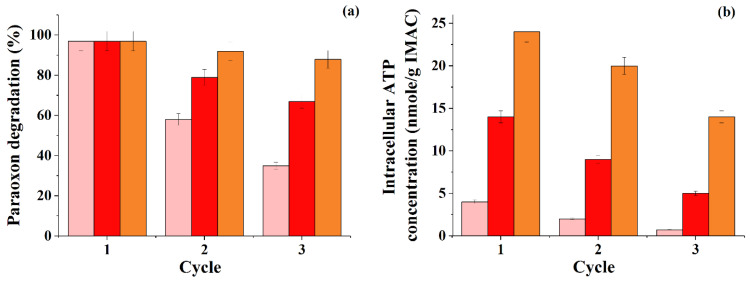
Multiple uses of IMAC in the paraoxon degradation (0.15 mm): (**a**) IMAC with cell concentrations of (■ 10% *w*/*w*), (■ 20% *w*/*w*), and (■ 30% *w*/*w*) and (**b**) changes in intracellular ATP concentration in repeatedly used samples of IMAC during paraoxon degradation.

The possibility of the long-term use of multicomponent cellular biosystems in the form of immobilized synthetic consortia, as a rule, is an important task for IMAC developers and a significant characteristic of the art biocatalysts themselves. An essential role in increasing the stability of the functioning of immobilized microorganisms during their prolonged use is played by their initial concentration inside the carrier granule. Using the example of cells of various bacteria immobilized in a cryogel of PVA, we previously showed that many microorganisms exhibit their maximum viability when their initial concentration inside the carrier granule is 30% instead of 10% (*w*/*w*) [30,36,37]. In this regard, at this stage of experimental work, when testing the effectiveness of the functioning of the IMAC, which contained *P. esterophilus* and *R. ruber* cells in a ratio of 2:1 in 50 mg granules and was used in the process of the multiple degradation of paraoxon (Figure 2), the total content of cell biomass in the granule (from 10% to 30%) was boiled, but at the same time, the total concentration of cells in the paraoxon medium was kept the same (20 g/L).

During the experiment, the total concentration of intracellular ATP in granules with IMAC was monitored since it was this parameter that most accurately allowed us to assess the overall level of metabolic activity of the cells of microorganisms in the polymer granule. The change in this characteristic indicated the total effect of various factors on the metabolic activity of cells in the consortium and, according to our extensive experience in applying such an ATP control to different biosystems, allowed us to adequately assess the situation with the state of immobilized cells as a whole [37,38].

The repeated use of IMAC containing 10% of the cell biomass during the degradation of paraoxon showed that with each subsequent cycle there was a decrease in the degree of degradation of paraoxon (Figure 2a). On the contrary, when using IMAC obtained by loading granules with 30% cell biomass, the degree of paraoxon degradation notably changed in several consecutive working cycles of the use of immobilized cells. Therefore, in the third cycle, the conversion speed of the pesticide for IMAC with 30% of the biomass in granules was 2.5 times higher compared to the IMAC in the form of PVA cryogel granules loaded with 10% cell biomass. It is obvious that the high concentration of cell biomass inside the PVA cryogel granules was the reason for the more stable functioning of the IMAC in an environment with a pesticide. It is possible that an increase in the concentration of immobilized cells per unit volume could contribute to the formation of a stronger “quorum response” in them and, hence, increased stability with prolonged use of the consortium.

The change in the specific concentration of intracellular ATP in granules with cells during the use of IMAC with a 30% content of bacterial cell biomass in granules testified that this variant of the consortium was significantly better than the other studied variants. It preserved the metabolic activity in the paraoxon degradation when IMAC was repeatedly used for this process (Figure 2b). In general, the main tendency to decrease the level of ATP in cells in all variants of IMAC whose metabolic states were controlled during the degradation of paraoxon in several consecutive work cycles, suggests that paraoxon, used by cells as the only carbon source, is not the best substrate for them.

It is interesting to note that such an observation was made in the process of the repeated use of IMAC in the degradation of paraoxon. When using individual bacterial cultures for the destruction of paraoxon, in the case of *P. esterophilus* cells, a gradual acidification of the medium occurred, at which the pH value of the medium decreased from 7 to 6.5, and in the case of *R. ruber* cells, a slight alkalinization of the medium was noted, which was accompanied by a slight increase in pH from 7 to 7.5 (Figure S1). In the case of the use of IMAC, in which both cultures were present, the pH value of the medium during the degradation of paraoxon was neutral, which may have had a favorable effect on the growth and development of cells of both microorganisms.

### 3.4. Degradation of Various OPPs and Hydrolysis of AHLs under the Action of IMAC Based on P. esterophilus and R. ruber Cells

**Figure 3:**
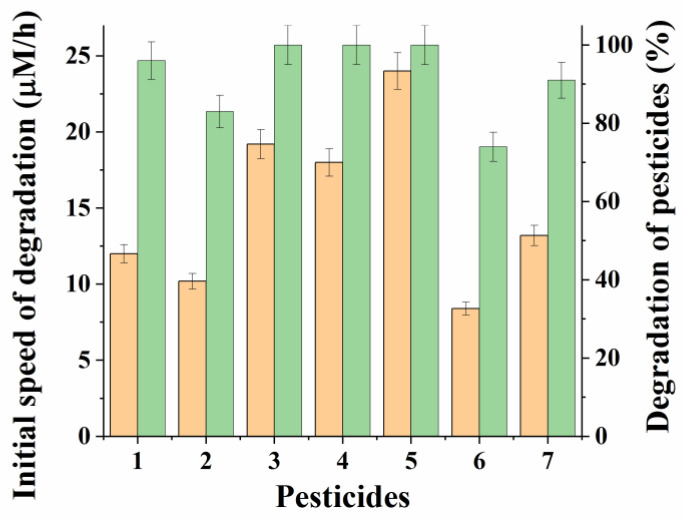
Degradation (■ initial speed and ■ degradation degree) of various OPPs (0.15 mM) by IMAC for 24 h: 1—parathion, 2—methyl parathion, 3—diazinon, 4—chlorpyrifos, 5—malathion, 6—dimethoate, 7—demeton-S-methyl.

The developed IMAC with a 30% concentration of cells in the PVA cryogel granules reduced in size and a mass ratio of “*P. esterophilus: R. ruber”* cells equal to 2:1 was used to investigate the possibility of using it for the degradation of various OPPs in the composition of a minimal medium (Figure 3 and Figure S2).

At the same time, as before in the experiments with paraoxon, the initial rate and degree of degradation of OPP injected into the medium at the same concentration (0.15 mM) were controlled. The process was observed, as before, for 24 h when IMAC was introduced into the studied media in a total concentration of cell biomass of 20 g/L. It turned out that the IMAC obtained on the basis of *P. esterophilus* and *R. ruber* cells carried out a very effective degradation of all the studied OPPs. At the same time, in most cases, the degree of degradation of pesticides in 24 h was more than 90%, and for diazinon, malathion, and chlorpyrifos it was 100%.

In view of the success of the obtained results in the degradation of OPP, the analysis of the catalytic activity of the obtained IMAC in the hydrolysis of various AHLs was of further special interest to us. This is a precursor since it is well-known that the lactonase present in the cells of *R. ruber* AC-1513D [19] hydrolyzes not only OPP but also different AHLs that differ in their structure.

At the same time, the induction of lactonase biosynthesis in *Rhodococcus* cells, as a rule, can be stimulated by the appearance and presence of certain lactone-containing compounds in the microenvironment of these bacteria [39], which in turn can actively synthesize *Pseudomonas* cells. For example, G(−) *Pseudomonas aeruginosa* cells secrete *N*-butyryl homoserine lactone (C4-AHL) and *N*-(3-oxododecanoyl)-homoserine lactone as QS signaling molecules [40,41]. Thus, some cells, synthesizing signaling molecules in the composition of IMAC to enter a stable state of QS, can synthesize AHL, while the latter can increase the level of synthesis of enzymes whose catalytic activity is directed against this and, in particular, on the hydrolysis of AHL and, along with this altogether, the general competition of cells for the availability of substrates leads to an increase in the degradation potential of the consortium. Checking the level of lactonase activity in immobilized cells could confirm these arguments.

An analysis of lactonase activity against different AHLs in IMAC taken after its direct formation and after its use for paraoxon degradation was carried out in comparison with immobilized individual *R. ruber* cells, and the concentration of *Rhodococcus* cells was the same in both cases (Table 2).

The obtained results showed that the level of lactonase activity for all the studied AHL molecules in the cells of IMAC increased almost proportionally, which indicated that the level of biosynthesis of enzymes capable of hydrolyzing AHL increased. It is also interesting to note that precisely in relation to those AHL (C4-AHL) molecules, which are known to be typical of *Pseudomanas* cells, one of the highest lactonase activities was detected in *Rhodococcus* cells and IMAC. At the same time, lactonase activity was maintained in IMAC (Table 2) after its repeated use for the degradation of paraoxon (Figure 2). Thus, the cells in the composition of such an IMAC were clearly in the QS state and showed mutual influence, which positively affected the results of OPP degradation.

## 4. Discussion

The detoxification of OPP can be realized using various physio-chemical approaches (oxidation, electrochemical methods, sonolysis, photocatalytic degradation, etc.) [42,43] and biological (enzymatic or/and microbial) [20] methods. The biological degradation of OPP with the participation of microorganisms looks more attractive from the ecological point of view since cells can effectively remove pesticides from contaminated sources and involve them in deep conversion through regular metabolism.

There are different approaches to the creation of synthetic bacterial cell consortia for the biodegradation of pollutants:

- The cultivation of natural consortia on certain media containing pollutants in order to accumulate increased concentrations of those cells that are able to exist in the proposed conditions, with the loss of those participants in these natural consortia who failed to cope with the proposed conditions. This method is often called the “adaptation and selection” of consortium members under the influence of a pressing pollutant [36,37]. As a rule, these are lengthy processes with an unpredictable and difficult to reproduce final version of the composition of the consortium members;
- The introduction of additional strains into natural consortia to enrich them with those cells that are the most active participants in the necessary process [36,37]. However, any natural strains are always difficult to maintain and reproduce after the identification of all the original participants.

Artificial consortia are practically devoid of these disadvantages, the development of which is becoming an integral part of the actively developing synthetic biology today. The most interesting consortia are those that consist of a small number of participants, are easily reproducible in their composition and characteristics, and are stable in their functioning, which is especially important when it is necessary to use such biosystems for the degradation of toxic compounds. Since competition between microbial cells is often an incentive to improve the characteristics of their overall functioning, the main task in creating such a biosystem is to find a balance and basic elements capable of maintaining such a balance for a long time. It is obvious that the elements of the cellular quorum play an important role in this balance. Species in a consortium can exercise both positive and negative control over each other’s activities by exchanging metabolic intermediates that either promote or reduce the metabolic activity of their neighbor.

It is known that during their growth, G(−) bacteria secrete AHL into the medium, which induces QS in these microorganisms when a certain threshold concentration is reached [44]. Lactonase biosynthesis in *R. ruber* cells can be induced by AHL secreted into its microenvironment by the G(−) bacteria *P. esterophilus*, which together with *Rhodococcus* cells, were introduced into the IMAC to degrade OPP. Thus, *Rhodococcus* cells compete with other bacteria for their viability.

*Rhodococcus* cells can use their enzymes, including lactonase, for the degradation of a wide range of pollutants and toxicants, including molecules derived from OPP [45,46,47]. The steps in the degradation of these compounds are often associated with an intermediate lactonase activity.

It is interesting that 3-oxo substituted-AHL molecules possess bactericidal influence on G(+) bacteria [48]. Indeed, G(+) bacteria may have developed AHL-degrading enzymes to protect themselves against the antibacterial activity of AHL molecules, thereby favoring their survival in the natural environment. Moreover, it appeared that lactone-containing molecules with an aliphatic branched chain on the lactone ring can be fully assimilated by *Rhodococcus* cells [49].

It is known that the cells of bacteria of the genus *Rhodococcus* possess a complex of enzymes that are able to degrade *p*-nitrophenol [50]. Apparently, *R. ruber* cells also possess certain enzymes that carry out the destruction of this pollutant (Table 1).

The joint functioning of these cells and *P. esterophilus* in one reaction system obviously leads to the fact that the degree of conversion of *p*-nitrophenol increases with the use of IMAC. This is clear evidence of the complex synergistic interaction of *R. ruber* and *P. esterophilus* bacterial cells in the process of paraoxon degradation.

Interestingly, in all the work carried out in all the experiments, the total concentration of cells in the medium did not change and was the same (20 g/L), but we consistently redistributed these cells inside the carrier granules. After finding the necessary combination of two cultures (*P. esterophilus* and *R. ruber*), their ratio inside the granules was changed (from 1:1 to 2:1), then the packing density of the cells inside the granules was tripled (from 10% to 30% of the biomass of the mixed cells), but the size of the granules was reduced (from 250 to 50 mg).

All these stages led to an increase in the rate of decomposition of the pesticide by 2.25 times compared to the initial use of the two-component consortia (Table 1, Figure 2) as well as to an increase in the same parameter by 4.5 and 16 times compared with the separate *P.esterophilus* and *R. ruber* cells. Paraoxon degradation occurred effectively over all three cycles when using 30% *w*/*w* IMAC, and this was confirmed by the data in Figure 2, while there was a decrease in the level of ATP concentration in the same IMAC sample, but this did not happen sharply, and it only happened twice. According to our extensive experience using ATP analysis to monitor the metabolic activity of various consortia [36,37,38,51], such a decrease in the level of ATP indicates that the biocatalytic system metabolically rebuilt itself under new operating conditions, but at the same time, cells retain a high level of viability and the ability to degrade the toxic substrate. Similar changes in ATP concentration were found in the other studied IMAC samples. However, unlike IMAC with 30% *w*/*w* cells in granules, in the other variants there was not only a decrease in the level of paraoxon degradation but a decrease in ATP by 3–4 times was also observed. The increased concentration of cells in the IMAC content certainly provided improved stability of the biocatalytic system functioning.

In such synthetic consortia containing G(+) and G(−) bacteria, cells control each other’s survival using two different QS systems, and their immobilization in a state of close contact at high cell concentrations creates good conditions for maintaining a quorum state in both cultures.

In general, a comparison of the results obtained in this work on the degradation of OPP using IMAC after several stages of its improvement with recent results known from the literature on the use of individual immobilized maximally active cells of microorganisms for similar biodegradation experiments, whose action is improved with the addition of enzymes that catalyze the conversion of OPP [24], clearly indicates their comparability in terms of the speed of the process and its effectiveness (the degree of degradation of pollutants).

## 5. Conclusions

In our case, when creating a synthetic immobilized and stabilized consortium, we used strains with OPP degradation activity, which was far from the highest level of the parameter. We did not want to demonstrate the record-breaking ability of individual cultures, but we pursued another goal in the study, namely, to show how natural processes can be used without the genetic improvement of cells to manage them and direct them to intensify and improve the necessary characteristics of the process, such as the speed and levels of biodegradation and the use of a wide range of substrates that increase the stability of a functioning synthetic biocatalytic system. At the same time, we performed several purposeful, scientifically based sequential actions that led us to significant success. In fact, by obtaining the possibility of 100% degradation of various OPPs and reusing IMAC, we managed to significantly increase the speed of the process compared to the individual cultures involved in IMAC using their quorum state and strengthening it by the co-inclusion of cells in a macroporous carrier during their immobilization.

## Figures and Tables

**Table 1:** Results of paraoxon or *p*-nitrophenol degradation (0.15 mM) under the action of immobilized bacterial cells or various variants of IMAC (20 g wet cells/L).

N	* Immobilized Microorganisms or Consortium (IMAC) Composition						Paraoxon				*p*-Nitrophenol		
							** V_0_(µM/h)	De(%)	Dt (%)	ID_PX_(%)	De(%)	Dt(%)	ID_NP_(%)
1	*Rhodococcus erythropolis*						2.0 ± 0.2	27.0 ± 1.3	27.0 ± 1.3	0	20.0 ± 0.9	20.0 ± 0.9	0
2	*R. ruber*						2.0 ± 0.2	34.0 ± 1.7	34.0 ± 1.7	0	13.0 ± 0.6	13.0 ± 0.6	0
3	*Pseudomonas* sp.						7.4 ± 0.2	78.5 ± 3.9	78.5 ± 3.9	0	68.0 ± 3.4	68.0 ± 3.4	0
4	*P. esterophilus*						7.2 ± 0.3	73.9 ± 3.6	73.9 ± 3.6	0	45.2 ± 2.2	45.2 ± 2.2	0
5							1.9 ± 0.1	36.1 ± 1.8	30.5 ± 1.5	5.6 ± 3.3	21.3 ± 0.9	16.5 ± 0.8	4.8 ± 0.2
6							4.7 ± 0.2	59.7 ± 2.9	50.4 ± 2.5	9.3 ± 0.4	47.3 ± 2.3	32.6 ± 1.6	14.7 ± 0.2
7							4.6 ± 0.2	63.6 ± 3.1	52.8 ± 2.6	10.9 ± 0.5	56.2 ± 2.8	44.0 ± 2.2	12.2 ± 0.6
8							7.2 ± 0.2	84.5 ± 4.2	53.9 ± 2.6	30.6 ± 1.5	63.6 ± 3.1	29.1 ± 1.4	34.5 ± 1.7
9							6.1 ± 0.2	80.4 ± 3.9	76.2 ± 3.8	4.2 ± 0.2	56.6 ± 2.8	56.6 ± 2.8	0
10							5.2 ± 0.2	75.4 ± 3.7	56.3 ± 2.8	19.1 ± 0.9	57.1 ± 2.8	40.5 ± 1.9	16.6 ± 0.8
11							3.8 ± 0.2	57.7 ± 2.8	46.5 ± 2.3	11.2 ± 0.5	41.8 ± 1.9	33.7 ± 1.6	8.1 ± 0.4
12							5.2 ± 0.2	60.4 ± 2.9	45.0 ± 2.2	15.4 ± 0.7	39.1 ± 1.9	26.1 ± 1.3	13.0 ± 0.6
13							5.5 ± 0.2	63.9 ± 3.1	59.8 ± 2.9	4.1 ± 0.2	54.9 ± 2.7	44.4 ± 2.2	10.5 ± 0.5
14							6.5 ± 0.2	78.0 ± 3.9	62.0 ± 3.1	16.0 ± 0.8	59.0 ± 2.9	42.0 ± 2.1	17.0 ± 0.8
15							5.2 ± 0.2	65.5 ± 3.2	53.3 ± 2.6	12.1 ± 0.6	46.4 ± 2.3	36.5 ± 1.8	9.9 ± 0.4

* ■ *R. erythropolis* ■ *R. ruber* ■ *Pseudomonas* sp. ■ *P. esterophilus* ** V_0_—the initial rate of paraoxon degradation (µM/h); De—the experimental degree of degradation (%); Dt—the theoretical degree of degradation (%); ID—improvement of degree degradation for Paraoxon (ID_PX_) and *p*-Nitrophenol (ID_NP_) correspondently (%).

**Table 2:** Lactonase activity of *R. ruber* AC-1513D cells and IMAC after its application in the degradation of paraoxon.

AHL	*R. ruber*, U/g Dry Cells	IMAC (U/g Dry Cells) After	
		* The 1st Cycle	The 3rd Cycle
*N*-Homoserine lactone hydrochloride	2.3 ± 0.1	4.0 ± 0.2	4.5 ± 0.1
*N*-butyryl-D,L-homoserine lactone	6.0 ± 0.2	11.6 ± 0.3	11.4 ± 0.4
*N*-hexyl-L-homoserine lactone	5.6 ± 0.2	9.8 ± 0.2	9.5 ± 0.3
*N*-(3-oxo-octyl)-L-homoserine lactone	6.2 ± 0.2	12.0 ± 0.4	12.4 ± 0.3
*N*-(3-oxo-decyl)-L-homoserine lactone	5.4 ± 0.1	9.2 ± 0.3	9.0 ± 0.4
*N*-(3-oxo-dodecanoyl)-L-homoserine lactone	5.0 ± 0.2	9.1± 0.2	9.2 ± 0.2

* See the multiple uses of the IMAC for paraoxon degradation in Figure 2.

## Data Availability

The data presented in this study are available by request from the corresponding author.

## Supplementary Materials

The following supporting information can be downloaded at: https://www.mdpi.com/article/10.3390/microorganisms10071394/s1, Figure S1: pH values of the medium used for paraoxon biodegradation by bacterial strains and IMAC (■—*P. esterophilus*, ■—*R. ruber*, and ■—IMAC) during the cell cultivation; Figure S2: Chromatograms of OPP degradation by IMAC.
