# Supplementary material for: “Unity and Struggle of Opposites” as a Basis for the Functioning of Synthetic Bacterial Immobilized Consortium That Continuously Degrades Organophosphorus Pesticides
Source: Microorganisms. 2022 Jul 11;10(7):1394. doi: 10.3390/microorganisms10071394 (PMC9317566; doi:10.3390/microorganisms10071394)
Supplement: Supplementary file 1 [file microorganisms-10-01394-s001.zip › microorganisms-1796279-supplementary.pdf]

Supplementary Materials

Microorganisms

"Unity and struggle of opposites" as a basis for the functioning of synthetic bacterial immobilized consortium that continuously degrades organophosphorus pesticides

Elena Efremenko[1]*, Nikolay Stepanov[1], Olga Maslova[1], Olga Senko[1], Aysel Aslanli[1], Ilya Lyagin[1]

[1]Lomonosov Moscow State University, Lenin Hills 1/3, 119991 Moscow, Russia;
elena_efremenko@list.ru (E.E.); na.stepanov@gmail.com (N.S.); olga.maslova.rabota@gmail.com (O.M.); senkoov@gmail.com (O.S.); ayselaslanli@mail.ru (A.A.); lyagin@mail.ru (I.L.);
* Correspondence: elena_efremenko@list.ru; Tel.: +7-495-939-3170; Fax: +7-495-939-5417

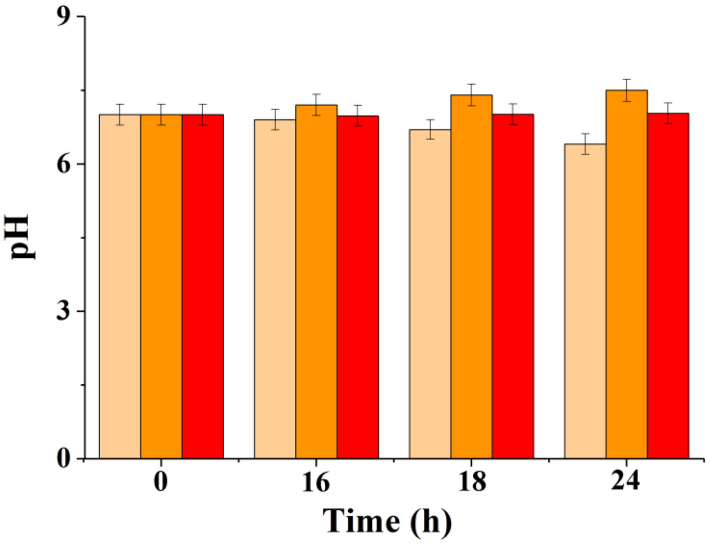

Figure S1. pH values of the medium used for paraoxon biodegradation by bacterial strains and IMAC (■ - *P. esterophilus*, ■ - *R. ruber*, ■ - IMAC) during the cell cultivation.

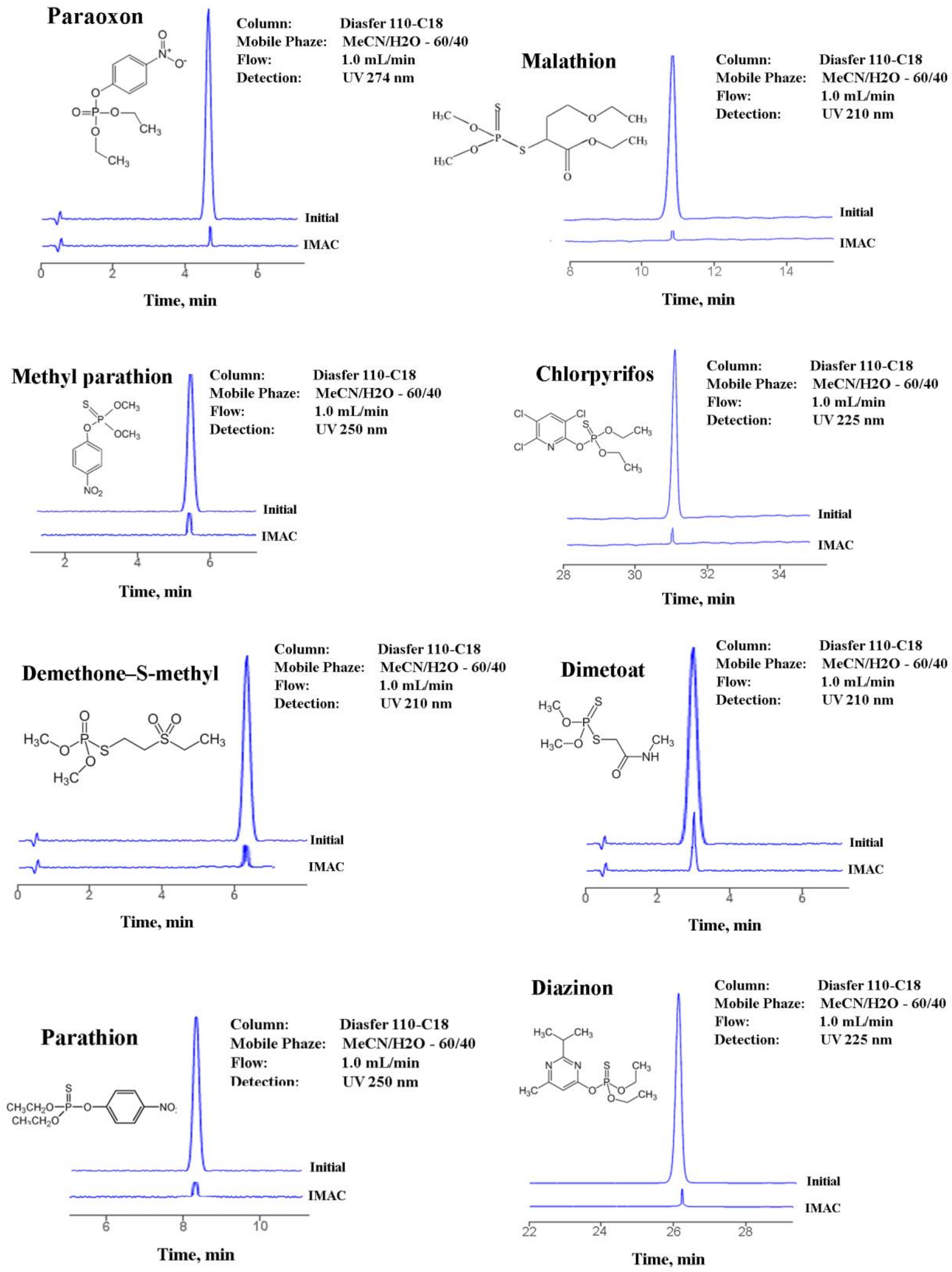

Figure S2. Chromatograms of OPP degradation by IMAC.
